# A qualitative review of implementer perceptions of the national community-level malaria surveillance system in Southern Province, Zambia

**DOI:** 10.1186/s12936-016-1455-7

**Published:** 2016-08-08

**Authors:** Lynne Lohfeld, Tokozile Kangombe-Ngwenya, Anna M. Winters, Zunda Chisha, Busiku Hamainza, Mulakwa Kamuliwo, John M. Miller, Matthew Burns, Daniel J. Bridges

**Affiliations:** 1 Bachelor of Health Sciences (Honours) Program, McMaster University, Hamilton, ON Canada; 2 Akros, Cresta Golfview Grounds, Great East Road, Lusaka, Zambia; 3 National Malaria Control Centre, Government of Zambia Ministry of Health, Lusaka, Zambia; 4 Malaria Control and Evaluation Partnership in Africa (MACEPA/PATH), Lusaka, Zambia

**Keywords:** Malaria elimination, Surveillance, Reactive case detection, Qualitative, Community health worker, Zambia

## Abstract

**Background:**

Parts of Zambia with very low malaria parasite prevalence and high coverage of vector control interventions are targeted for malaria elimination through a series of interventions including reactive case detection (RCD) at community level. When a symptomatic individual presenting to a community health worker (CHW) or government clinic is diagnostically confirmed as an incident malaria case an RCD response is initiated. This consists of a CHW screening the community around the incident case with rapid diagnostic tests (RDT) and treating positive cases with artemether-lumefantrine (AL, Coartem™) in accordance with national policy. Since its inception in 2011, Zambia’s RCD programme has relied on anecdotal feedback from staff to identify issues and possible solutions. In 2014, a systematic qualitative programme review was conducted to determine perceptions around malaria rates, incentives, operational challenges and solutions according to CHWs, their supervisors and district-level managers.

**Methods:**

A criterion-based sampling framework based on training regime and performance level was used to select nine rural health posts in four districts of Southern Province. Twenty-two staff interviews were completed to produce English or bilingual (CiTonga or Silozi + English) verbatim transcripts, which were then analysed using thematic framework analysis.

**Results:**

CHWs, their supervisors and district-level managers strongly credited the system with improving access to malaria services and significantly reducing the number of cases in their area. The main implementation barriers included access (e.g., lack of rain gear, broken bicycles), insufficient number of CHWs for programme coverage, communication (e.g. difficulties maintaining cell phones and “talk time” to transmit data by phone), and inconsistent supply chain (e.g., inadequate numbers of RDT kits and anti-malarial drugs to test and treat uncomplicated cases).

**Conclusions:**

This review highlights the importance of a community surveillance system like RCD in shaping Zambia’s malaria elimination campaign by identifying community-based infections that might otherwise remain undetected. At this stage the system must ensure it can meet growing public demand by providing CHWs the tools and materials they need to consistently carry out their work and expand programme reach to more isolated communities. Results from this review will be used to plan programme scale-up into other parts of Zambia.

## Background

Globally about 3.3 billion people are at risk of developing malaria each year. The burden is greatest in the WHO African Region where about 90 % of all malaria deaths occur, with 78 % in under-five children [1]. Of 106 countries and territories with malaria transmission in 2000, 102 have reversed the incidence of malaria [2], and 55 are on track to meet the roll back malaria and World Health Assembly targets of reducing malaria case incidence rates by 75 % by 2015 [1].

Despite such progress, the 55 countries with decreases of >75 % in malaria incidence accounted for only 13 million (6 %) of the total estimated cases. In part this is because countries with the largest number of cases have made slower progress and have poorer quality surveillance data, particularly in sub-Saharan Africa [1]. In sub-Saharan Africa, while progress has been mixed, the number of severe malaria cases and malaria deaths have reduced significantly [2]. Some areas of southern and eastern Africa have reached transmission rates low enough to consider embarking on malaria elimination campaigns that would completely interrupt local mosquito-borne transmission [3–5]. The Zambian government with assistance from several partners is working to create malaria-free zones through a number of initiatives. These include mass distributions of long-lasting insecticide nets, indoor residual spraying campaigns and effective case management at facility and community level. More novel approaches are being attempted under operational research to reduce community parasite reservoirs that remain in the presence of vector control methods, including mass test and treat or mass drug administration campaigns in areas of low to moderate transmission [6].

Diagnosing and treating malaria cases and infections are critical to treatment strategies for surveillance, control and elimination. From the perspective of the health system, malaria case or infection detection can be passive (the health system does not seek out individuals but waits for symptomatic individuals to present at a health facility), active (the local health system actively searches for symptomatic or asymptomatic infections in people who may otherwise not seek care at a health facility), or reactive (the local health system screens for additional infections around each confirmed incident malaria case who was confirmed after presenting to a health facility).

All of these approaches have strengths and weaknesses. For example, passive detection may miss cases in people who are asymptomatic or choose not to access the public health system due to factors such as distance or perception [7–9]. Campaigns based on active detection can be expensive over the short term and unnecessary in some situations where transmission is very low [10, 11]. Reactive case detection (RCD) is based on the premise that malaria cases are spatially and temporally clustered around incident cases so targeting community members living with or near incident cases can help avert additional transmission [12, 13]. Using RCD to identify a potential cluster of transmission may reduce the likelihood of sustaining low levels of transmission that can lead to larger outbreaks.

The current Zambian RCD system grew out of a need to more efficiently apply control methods to low transmission settings. It was originally developed in the capital city, Lusaka, a densely populated, urban setting where malaria transmission was known to be very low (from cross sectional survey sampling), and the vast majority of cases were thought to be imported. By tracking cases at the community level, data were generated that pointed to the value of limiting vector control measures to specific focal areas where transmission was still occurring [14].

As national surveillance efforts and interest in elimination grew, developing a scalable malaria surveillance system to track community-level contributions to malaria cases became increasingly necessary because research has highlighted the need for greater access to care among many Zambians, particularly in rural areas [15]. Since 2011 the RCD system was expanded into rural areas in 17 districts in Southern, Central and Western Provinces to increase case management capacity, enhance surveillance granularity, and identify areas of residual transmission [16]. This system is the largest-scale RCD operation in sub-Saharan Africa of which the authors are aware.

Briefly, in the Zambian rural RCD system, health care providers use rapid diagnostic tests (RDT) or microscopy to test symptomatic community members presenting at rural health posts and health centres to passively detect malaria infections. Once a confirmed symptomatic incident (index) case has been identified, Community-based CHWs are then dispatched to test and treat individuals living within approximately 140 m of the index case household using RDTs and treating RDT-positive cases with ACT. The RCD response radius was determined by analysing data from a large-scale mass test and treat programme in Zambia in which the investigators learned that the probability of identifying an infection beyond 140 m of an incident case was no greater than the chance of finding an infection in the general population [16]. The expanded RCD system increases the number of people included in malaria surveillance, testing and treatment while providing the on-going support of personnel working to control or eliminate malaria in a sustained way. This paper seeks to identify the perceptions and barriers to acceptance of the rural RCD system in four districts of Southern Province, Zambia. Such information will be used to identify barriers and how to overcome them to ensure the RDT programme can successfully expand into additional areas of the country.

## Methods

### Study site

**Fig. 1:**
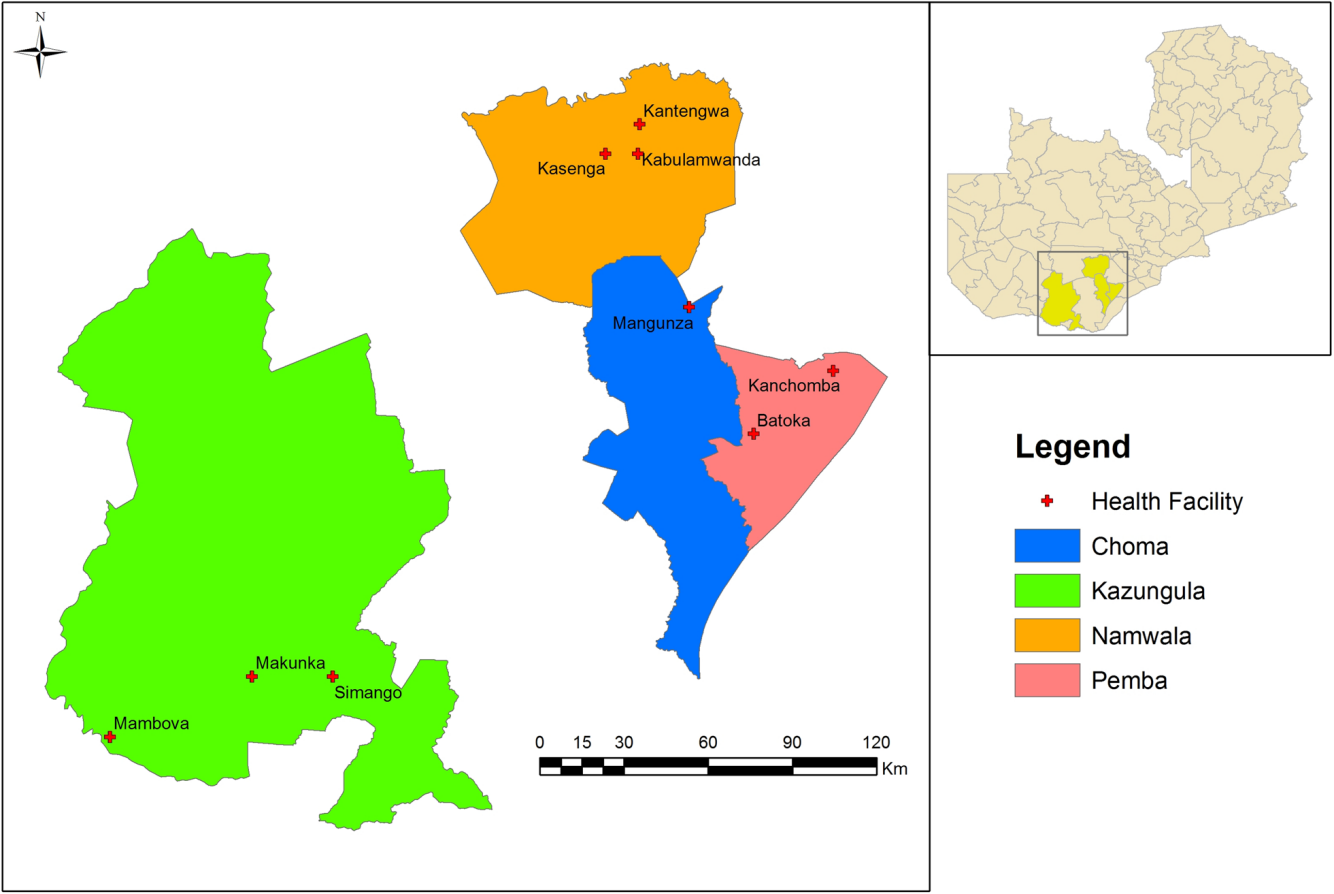
Map of case study area in Zambia, including the location of each of the health facilities enrolled in this study

Choma, Pemba, Namwala and Kazungula Districts in Southern Province were selected for review because they represent three waves of CHW training, each with slight modifications to content and/or duration that might affect the ability of CHWs to carry out the programme and other implementation barriers (Fig. 1). The population of these districts totaled 480,647 as of 2012 and are served by 72 rural health centres and 576 affiliated rural health posts (1–16 posts per health centre).

### Sampling

Nine rural health clinics with year-round access were enrolled (three each from Namwala and Kazungula Districts, two from Choma District and one from Pemba District, which became an administratively independent district from Choma after the start of the RCD Programme) that carried out the government’s RCD Programme. The facilities were classified as more versus less successful in implementing the programme using criteria in order to determine if participants’ description of barriers to a successful programme varied based on the facility’s level of implementation success. Criteria included the reporting rates and completeness of data collected by CHWs, level of CHW engagement, and the extent to which the local population consulted programme CHWs level of community coverage (see Table 1).

**Table 1:** Distribution of rural health facilities carrying out reactive case detection of malaria cases in communities of Southern Province, Zambia

District	Number of rural health facilities by level of functioning^a^	
	Higher	Lower
Choma	1	1
Pemba	0	1
Namwala	2	1
Kazungula	2	1
Total	5	4

^a^Ratings were based on consensus by Akros personnel overseeing the national malaria reactive detection programme based on the following characteristics: data reporting rates and completeness by CHWs, level of CHW engagement in programme activities, and extent to which the local population consults programme CHWs (level of community coverage)

### Data collection

A team of nine fieldworkers who spoke English, Chitonga and/or Silozi completed a four-day training course in qualitative research and fieldwork skills before starting to collect interview data. Interview guides were developed with input from the literature and expert opinion gathered through the Delphi Technique [17]. The fieldworkers translated three parallel guides, one per respondent group (district health offices or District Health Officers (DHO), on-site supervisors, and CHWs), from English to Chitonga and Silozi and then refined through group review during training. The guides consisted of open-ended questions and probes to elicit information about perceived programme impact, training and supervision of CHWs, programme barriers, incentives for CHWs and supervisors, and recommendations for overcoming barriers and scaling up the programme, as well as questions on demographic characteristics.

Data collection began immediately after the training course ended in September 2014. Interviewers obtained oral informed consent from each participant at the start of each interview. To ensure accuracy, oral data were digitally captured with participants’ permission and in written field notes. Local ethical approval was obtained from ERES Converge (Ref 2014-Mar-002) before starting the project.

### Data analysis

A verbatim copy or transcript of each audio file was created in the language of the interview and then translated into English. Data were analysed using thematic framework analysis [18] that incorporated both deductive categories (questions from the interview guides) and inductive findings (unanticipated comments) that enriched the study. Separate tables were created for each stakeholder group (DHO staff, CHW, and Clinic Supervisor) that displayed the data sorted by theme and subthemes with quotes to illustrate the points raised by respondents. Because of the small sample size there was a risk that individual participants could be identified, therefore only the group identifier (DHO, Clinic Supervisor or CHW) was used to indicate the source of each quote [19].

To distinguish between the study authors’ voice and that of study participants, transcript excerpts are printed in italics with ellipses (…) to indicate where words were removed and non-italicized text in square brackets to show where they were added to improve comprehension without altering meaning.

## Results

Data analysis revealed no clear patterns associated with the district or health facility. Data were therefore combined across sites before analysis, while noting similarities and differences across the three respondent groups.

### Participants

Twenty-two key informant interviews were performed with a cross section of staff working in the Zambian RCD Programme, including four interviews with district health office staff members, nine with CHWs and nine with clinic supervisors. Depending on the preference and availability of study participants, each interview included from one to three staff members.

### Perception about programme impact

Overall, programme staff reported that they thought highly of the RCD programme and its ability to promote national development through malaria elimination. As one CHW noted, *“We appreciate the programme because from the time they trained us, the country is developing and malaria is declining.”*

The vast majority of participants indicated they believed that malaria was on the decline in their catchment area and no longer a major cause of morbidity or mortality. As another CHW explained, *“nowadays we test 20–30 people a day and don’t find any cases.”* Reasons people gave for the decline in malaria cases were successful vector control from such measures as the sustained use of long-lasting insecticide-treated nets and better case management including the RCD programme (*“The numbers are down due to the programme … it’s a success because RDT*-*positive cases are treated right away and managed well. Over three*-*quarters of such cases are responding to treatment*—Supervisor; *It has worked very well…70* *% of malaria cases are treated at the community level and only 30* *% at the clinic, so the CHWs have become very important*—DHO Staff). Public education or ‘sensitization’ of local residents to malaria causation, prevention and treatment was also noted as a reason for fewer cases (*If the CHW sees a high number of* [malaria] *cases he tells the headman to call a meeting for education*—CHW; *We sensitize communities year*-*round in health facilities and by CHWs when they are in an area”*—DHO staff).

A small proportion of respondents thought that the number of malaria cases had not declined because there is an annual seasonal increase (“[The numbers] *may be down now but in the rainy season it increases”*—Supervisor), geographical barriers to accessing care (*“We still see malaria in hard*-*to*-*reach areas, more than two cases a week”*—DHO staff), the existence of imported cases (*“We’re at a border…* [where many people] *pass through. They might be the ones who cause malaria”*—CHW) and increased sensitivity of the programme’s surveillance system used to detect malaria cases. As one DHO staff person explained: *“Since theprogramme came on board, we’ve been seeing more cases* [and it appears that] *incidence is going up. I think it’s because the CHWs are doing a lot of work and we’re actually seeing the truth: now we can track numbers in our programme and see what’s happening in the community* [as well as in the clinics].”

### CHW training

Most CHWs described the initial training that they received as effective. One Supervisor explained that *“before the training we had a large number of* [malaria] *cases, but ever since the numbers have declined.”* When asked about the need for additional training, many participants suggested providing both refresher courses for existing CHWs and another round of training for new CHWs: “([You should train more CHWs] *so each village has its own … some CHWs have big catchment areas and if they have five* [re]*active cases* [to follow up] *it’s too much work”*—CHW; *The population is growing, and of those trained some have died or given up. So we have a shortage* [of CHWs]. *“If we train new ones they can replace those CHWs”*—DHO Staff). Some respondents also thought it was important to expand training to include other health personnel such as environmental health and lab technicians, medical officers, and nurses working in rural health centres because *each staff person should know about RDT and reactive case finding. Consider it an orientation* (Supervisor). Suggested topics for training included information on referrals, documentation and registries, how to provide health education and counseling to patients, and information needed by CHWs hired specifically for the malaria RCD programme to help them address other health problems: “[We want to learn] *how to diagnose and treat other ailments … if you see an RDT*-*negative person who is sick with fever and vomiting, you want to help. It’s not enough to just refer them to the clinic because some people can’t get there”* (CHW).

There was a marked difference of opinion regarding the ideal composition of training classes. One view was it is helpful to bring people from various health centres together in a central location *so everybody shares their experiences* (Supervisor), whereas other people thought it would be better to offer separate training sessions for groups of CHWs working in areas with similar levels of malaria burden or community resistance (*We went for training with people from areas with more malaria cases* [than we have] *and communities refusing to have their blood taken… so the course was not very helpful*—CHWs).

### Barriers to programme success

Participants selected the three biggest implementation barriers for the programme from a list of nine options. Over half of all respondents identified the following four issues: inaccessibility due to flooding, lack of community confidence in CHWs’ ability to address diseases other than malaria, lack of community willingness to visit CHWs for malaria testing, and lack of motivation by CHWs. Lack of community/district ownership of the programme and lack of coordination between health clinic staff and CHWs were least often chosen as barriers (Table 2).

**Table 2:** Rank-ordered list of barriers to successful reactive case detection in sample of rural health centres of Southern Province, Zambia by respondent group

Problem	Total (n = 22)	DHOs (n = 4)	Supervisors (n = 9)	CHWs (n = 9)
	Rank no. (%)	Rank no. (%)	Rank no. (%)	Rank no. (%)
Inaccessible areas during the rainy season	*1st 16 (72.7)*	3rd 2 (50.0)	*1st 5 (55.6)*	*1st 9 (100.0)*
Lack of community confidence in CHWs to deal with other diseases besides malaria	2nd 15 (68.1)	*1st 4 (100.0)*	*1st 5 (55.6)*	3rd 6 (66.7)
Community not willing to visit CHWs for malaria testing	3rd 13 (59.1)	3rd 2 (100.0)	2nd 4 (44.4)	2nd 7 (77.8)
Lack of motivation for CHWs in the programme	4th 12 (54.5)	2nd 3 (75.0)	1st 5 (55.6)	4th 4 (44.4)
Stock out of commodities	5th 11 (50.0)	2nd 3 (75.0)	4th 2 (22.2)	3rd 6 (66.7)
Lack of feedback to CHWs and health facilities to let them know how they are performing	6th 8 (36.3)	4th 1 (25.0)	3rd 3 (33.3)	4th 4 (44.4)
Lack of seriousness by CHWs to carry out follow ups	6th 8 (36.3)	4th 1 (25.0)	2nd 4 (44.4)	5th 3 (33.3)
Lack of coordination between health clinic staff and CHWs	7th 5 (22.7)	4th 1 (25.0)	4th 2 (22.2)	6th 2 (22.2)
Lack of community/district ownership of programme	8th 1 (4.5)	5th 0 (0.0)	5th 0 (0.0)	7th 1 (11.1)

Highest ranked issue(s) is shown in italics

Suggestions to address the most pressing barrier, the lack of access to communities due to flooding, were to provide CHWs with rain gear or to boats. As one supervisor explained, *we have two hard*-*to*-*reach fishing areas and in the rainy season it is hard to get there. Cars can’t go there due to floods. From January to early June we don’t even go there for* [planned] *outreach!* Another idea was to expand the roster of CHWs to include those who live in flooded villages because they would not need to travel far in inclement weather.

DHO staff thought the lack of community confidence in the programme CHWs is because villagers know the volunteers are only able to test and treat simple cases of malaria and must refer other problems to their local health clinic whereas earlier government-sponsored CHWs could address a wide range of issues. Some supervisors also noted that because CHWs are seen as ‘village doctors’ community members are frustrated at the lack of help for non-malaria cases. This view was shared by CHWs, some of whom expressed a strong desire to be trained to diagnose and treat several conditions as noted by one worker: *“If a person is shivering and vomiting but the RDT is negative, it’s not good to leave the person without anything other than a referral to the clinic. They see that people who are RDT*-*positive get Coartem but that there’s nothing for them … they should also give us drugs so that we can help people without their going to the clinic.”*

Participants also provided reasons why they thought villagers were not accessing the CHW service. Frequently, this reticence was attributed to CHWs lacking testing kits or anti-malarials. As one CHW explained, the lack of community interest in being tested *is the biggest problem that we have. We’ve told staff at the health centre to tell people to come see us, but* [they don’t come] *because of the lack of kits.* During the period that interviews were conducted, there were RDT or anti-malarial stock outs at the national or local, i.e. health facility, level suggesting that commodities were not being released by to the CHWs. A situation that has previously been documented [20].

The lack of motivation on the part of some CHWs was linked to many CHWs feeling their community service went unrecognized. As one CHW commented, *We leave our family work* [as farmers] *and* [instead] *work for the community but at the end of the day we don’t get anything as appreciation for the work we do. We wish the government would do something for us because we are reducing the workload for nurses. At least* [they should give us a little money so] *we can buy soap!* Others noted they felt unappreciated because promises made by programme administrators, such as a mobile phone or monthly allotment of phone credit for each CHW, had not been kept.

### Incentives

Respondents discussed motivators for staff involved in the RCD programme. A few CHWs noted they were motivated by intrinsic incentives such as knowledge gains (*because I’m a CHW I know more about malaria*) but this may be because CHWs are given a small amount of money for each day they attend off-site training courses. Some participants explained they were motivated to carry out programme activities because they receive material needed to do this work, such as access to a bicycle that is given to the CHWs outright after completing 2 years with the programme. A few supervisors suggested that giving them bicycles would help improve the programme because they could more easily make supervisory visits to CHWs in the field.

Participants also identified programme disincentives. These include the lack of reliable supply of programme-related commodities such as RDT kits and AL, and delays in receiving replacement mobile phones or bicycle parts. By far the biggest problem noted by CHWs was the lack of stipend or financial support for the volunteers. As a DHO staff member noted, *There are some CHWs who have given up because they are looking for a permanent job or just stopped due to lack of incentives*—*reimbursement*—*because they have families to feed*. The vast majority of CHWs mentioning payment reported wanting just a small token payment either as acknowledgement of their work (*We’d like a little bit of money*—*not enough to deposit in the bank but just to show appreciation*—CHW) or to purchase simple supplies such as soap (*As change agents we touch dirty things, so we need soap*—CHW). Supervisors also commented on the importance of token payments to CHWs, explaining they are farmers who essentially have abandoned their family farms in order to carry out programme activities. CHWs could use small payments to either buy essentials, such as sugar, oil or foodstuffs, or to pay day laborers to help with farming activities.

Other incentives identified by programme staff include money for refueling clinic motorcycles so supervisors could visit CHWs in distant villages, and expanded kits for CHWs (*We need more drugs in our kit so we can treat the basic ailments in RDT*-*negative people and not just malaria*—CHW). In addition, more direct supervision and feedback would be helpful to CHWs, one of whom explained, *Tell them to encourage us. Yes, it’s true we’re* [just] *volunteers but people work better with motivation.*

### Recommendations made by programme staff

Overall the programme was generally perceived as beneficial. Prior to the programme RDT and treatment for malaria were available as an out-of-pocket item in private pharmacies in towns and cities, and free-of-cost at government health clinics and regional hospitals. The RCD system brought malaria care to isolated rural communities while enhancing the granularity of the national surveillance programme that can provide evidence needed to map malaria hotspots and health care needs at the sub-catchment level.

Key issues noted by programme staff as needing to be addressed prior to scaling up the programme include obtaining additional funding to expand the role of CHWs, addressing community concerns to increase local acceptance of CHWs to test and treat malaria, expanding marketing of the programme and improving coordination among the Government of the Republic of Zambia and its partners to better ensure an uninterrupted supply chain of essential commodities at the local level.

## Discussion

Both integrated community case management (iCCM) and many disease-specific programmes in low- and middle-income countries rely on CHWs to reduce the number of maternal and childhood deaths in hard-to-reach communities [21, 22]. Such programmes are built around task-shifting away from nurses at health centres to CHWs in the community. Because this cadre of workers is less well trained than nurses it is important that they receive adequate training and support in order to ensure good access, coverage, and quality of health services at a reasonable cost to the Government [23, 24]. In addition, it is important to identify bottlenecks to community acceptance of, and incentives for CHWs to maintain programme operations.

In this review of the RCD system to control malaria in Southern Province, Zambia, participants reported that the programme had reduced the number of malaria cases and outpatient case load in the study area, as well as improved efforts to locate malaria hotspots at the sub-catchment level. However, research has shown that initial improvements are often followed by high rates of CHW attrition that threaten programme sustainability and scalability [24].

Interviewed participants identified several problems with programme implementation ranging from the lack of community acceptance, to inadequate support to help the volunteers carry out their duties. This resonates with findings from a report on key evaluation questions and indicators identified by iCCM experts [25] that highlighted the need to promote community satisfaction with CHWs, high levels of service uptake, and strategies to maintain the motivation, retention, training, and supervision of CHWs.

One of the barriers participants identified was the difficulty CHWs face travelling to the community, particularly when the area is flooded in the rainy season. This matches the findings from a report on challenges to iCCM programmes in sub-Saharan Africa [26]. Suggestions the authors made to address this issue included providing CHWs with raingear to protect themselves and their equipment, access to canoes, and expanding the cadre of workers to include CHWs living in flood-prone areas.

During the interviews, it became clear that community members in the programme catchment area often bypassed CHWs in favour of having malaria tests done at the local health centre. This was attributed to disappointment that the CHWs were not trained and equipped to address a wide range of health problems, as had been earlier done in rural Zambia. Health extension worker bypass is still common in Ethiopian iCCM programmes [27]. Reports on how to strengthen CHW-based programmes suggest harmonizing the role and training of CHWs across all programmes in an area [28–30]. However, this increases the possibility of high patient loads and greater expectations of CHWs which can negatively impact an RCD programme, as was the case in Uganda [31].

Studies have shown that inadequate supervision and inadequate incentives frequently reduce CHW motivation and retention [32]. In this programme review, CHWs indicated they wanted more frequent and useful feedback from their direct supervisors. There are challenges to providing such supervision, including travel costs and logistics, supervision being treated as an add-on rather than a core element of medical personnel job descriptions, and the lack of training and support for supervisors. Some programmes, including this one, have tried to address this by providing mobile phones [33] or expanding supervisory roles to include groups, peers, and communities [29].

Perhaps the key issue is how to motivate CHWs to carry out their responsibilities and remain in a programme once trained. The common assumption that incentives refer mainly to payment and promotion is unfounded. In addition to financial benefits that accrue to individuals (direct incentives) such as salary, allowances, bonuses, and reimbursement of costs, there are also non-financial incentives including job satisfaction, autonomy, supportive supervision, manageable workload and professional development. Indirect incentives accrue to the system as a whole and exist at the level of the health care system and the community. There are also complementary or demand-side incentives such as appreciation from other health care workers and community members [29, 34, 35].

Efforts to strengthen programmes using CHWs or other community-based volunteers are based on identifying and addressing bottlenecks. One useful framework separates them by level [36]. The three non-systemic levels, community or household, health service delivery, and health sector policy or strategic management, are generally targeted for programme improvements. Other authors focus on supply- and demand-side barriers such as commodity stock outs, the lack of payment and longer-term career possibilities that can affect the deployment and availability of CHWs [37–39].

### Strengths and weaknesses

An important step in creating a sustainable and scalable programme is to periodically review processes and outcomes. To ensure high-quality reliable data this study used an outside team of trained fieldworkers led by an experienced qualitative researcher and programme investigator. Findings were reported anonymously so that participants would be more likely to discuss issues and barriers, while data were collected from multiple sources (triangulation). The fact that many of the barriers to programme implementation have also been identified by other investigators further strengthens the credibility of the findings presented here. In addition, interviewing rather than quickly surveying participants gave them a sense of being heard, which can enhance ownership and participation in future efforts to improve and evaluate the programme.

Based on the assumptions underlying qualitative case studies—that pinpointing differences across more versus less successful programmes can help highlight barriers and effective solutions for them—programme managers were asked to identify clinic characteristics and then categorise sites by level of performance. After finding no difference in data collected from different sites all data were combined for final analysis. It is unclear why no site differences were found, but could be related to the indicators chosen to collect. This may be explored in later evaluations of the programme.

## Conclusions

Study findings indicate that the Zambia RCD programme is already part of the accepted arsenal of tools to combat malaria in Southern Province. A key component of the programme is task-shifting from nurses to CHWs, which is impeded by such barriers as reduced access to communities flooded during the malaria season, a shortage of CHWs, the lack of community understanding of CHWs’ role and mandate, and the need for consistent support, supervision and incentives. Efforts to address these problems should be included in larger platforms of change designed to strengthen the Zambian health care system, including its malaria surveillance and monitoring programme.

## Abbreviations

ACT: artemisinin-based combination therapy
CHW: community health worker
DHO: district health officer
iCCM: integrated community case management
RCD: reactive case detection
RDT: rapid diagnostic test
